# Effectiveness of exercise therapy on chronic ankle instability: a meta-analysis

**DOI:** 10.1038/s41598-025-95896-w

**Published:** 2025-04-05

**Authors:** Chengcheng Zhang, Zhenzhou Luo, Dingwei Wu, Jie Fei, Tianpei Xie, Min Su

**Affiliations:** 1 The Fourth Affiliated Hospital of Soochow University, Suzhou, 086-215000 China; 2 Department of Orthopedics, Fujian Medical University Union Hospital, Fuzhou, 086- 350001 China; 3 The Affiliated Jiangning Hospital of Nanjing Medical University, Nanjing, 086- 210000 China

**Keywords:** Exercise therapy, Chronic ankle instability, Meta-analysis, FAAM, SEBT, Orthopaedics, Rehabilitation

## Abstract

Lateral ankle sprains are one of the most common musculoskeletal injuries. Up to 70% of individuals who sustain lateral ankle sprains develop chronic ankle instability (CAI). Exercise therapy is considered an effective treatment for patients with CAI. This meta-analysis investigated the efficacy of exercise therapy in CAI patients by reviewing 15 randomized controlled trials (RCTs) involving 586 participants. Databases including PubMed, EMBASE, Cochrane Library, and Web of Science were searched from inception to September 13, 2024. The Cochrane Risk of Bias Tool was used to assess study quality. Meta-analysis, sensitivity analysis, and publication bias analysis were conducted using RevMan 5.3.0 and Stata 18.0 software. The Grading of Recommendations, Assessment, Development, and Evaluations (GRADE) approach was applied to evaluate the quality of evidence. Main outcomes were assessed using the Foot and Ankle Ability Measure (FAAM) and the Star Excursion Balance Test (SEBT). The results demonstrated that exercise therapy significantly improved FAAM-S (MD = 7.98, CI: 4.11 to 11.86, *p* < 0.0001, I² = 30%). Long-term exercise therapy (over 4 weeks) significantly enhanced FAAM-A (MD = 10.95, CI: 6.60 to 15.29, *p* < 0.00001, I² = 0%) and dynamic balance ability of ankle joint (SBET-A: MD = 4.83, CI: 1.04 to 8.63, *p* = 0.01, I² = 62%; SEBT-PM: MD = 6.93, CI: 2.37 to 11.48, *p* = 0.003, I² = 69%; and SEBT-PL: MD = 8.98, CI: 2.66 to 15.29, *p* = 0.005, I² = 86%). After categorizing by exercise type, the results indicated that strength training was more effective in improving SEBT-PL (MD = 8.15, CI: 6.09 to 10.21, *p* < 0.00001, I² = 0%), joint mobilization was more effective in improving SEBT-A (MD = 7.65, CI: 4.93 to 10.37, *p* < 0.00001, I² = 0%), and proprioceptive training was more effective in improving SEBT-PM (MD = 10.46, CI: 5.27 to 15.65, *p* < 0.0001, I² = 33%). In conclusion, long-term, multifaceted exercise therapy demonstrates superior rehabilitation efficacy for patients with CAI. Personalized treatment plans, informed by SEBT assessment results, should prioritize targeted interventions such as joint mobilization, strength training, or proprioceptive training. This approach holds significant theoretical and practical value for optimizing CAI treatment strategies and enhancing patient outcomes.

**Supplementary Information:**

The online version contains supplementary material available at 10.1038/s41598-025-95896-w.

## Introduction

The ankle joint is the most frequently injured major weight-bearing joint in the human body, with ankle sprains being the most prevalent type of injury^1^. It primarily refers to injuries of the medial triangular ligaments surrounding the ankle joint, including the tibionavicular ligament, tibiocalcaneal ligament, anterior tibiotalar ligament, and posterior tibiotalar ligament, as well as the lateral collateral ligaments, including the calcaneus fibular ligament, anterior talofibular ligament and posterior talofibular ligament^2,3^. Once an initial ankle sprain occurs, individuals may experience chronic ankle instability (CAI), which is often characterized by a sensation of ankle instability, recurrent ankle injuries, and persistent symptoms such as pain, weakness, or reduced range of motion (ROM) in the ankle^4^.

Lateral ankle sprains are among the most common musculoskeletal injuries. In the United States, over 2 million cases occur each year, with approximately 73% classified as lateral ankle sprains, while around 25% are medial ankle sprains^5^. It is estimated that between 50% and 70% of patients do not receive appropriate rehabilitation following the initial injury, which can contribute to the development of CAI^6^. Lateral complex ligament injuries producing recurrent inversion sprains were termed lateral chronic ankle instabilities (LCAI); deltoid ligament lesions causing medial giving away sensation or pain received the medial chronic ankle instability (MCAI) nomenclature^7^. For individuals with CAI, their physical activity and quality of life may be significantly affected. The decline in walking function not only affects their ability to work but may also result in significant indirect costs, thereby negatively impacting their economic status.

Exercise therapy, also known as exercise rehabilitation, is a rehabilitative treatment modality that employs equipment, manual techniques, or the patient’s own strength to promote the recovery of both global and localized motor and sensory functions^8^. There is evidence to suggest that exercise therapy is a first-line option for many chronic diseases^9^. Compared to conventional care, exercise rehabilitation can reduce the risk of recurrent ankle sprains; however, there is currently insufficient data to determine the optimal components of exercise-based interventions^10^.

In recent years, numerous studies have demonstrated that exercise training is an effective treatment for CAI, with a significant emphasis on balance training^11,12^. A systematic review has indicated that balance training has a notable therapeutic effect on CAI; however, it did not evaluate the efficacy of other exercise therapy^13^. Furthermore, during the analysis, the study included multi-arm RCTs with repeated control groups, which may affect the validity of the results. The studies included in this review had control groups that incorporated physical therapy and other exercise modalities, making it impossible to exclude the potential impact of exercise in the control group. The research by Wang et al. only provided a meta-analysis framework. Therefore, the consistency of the effects of balance training compared to other exercise therapy remains unclear^14^.

This meta-analysis examines the effectiveness of exercise therapy in the treatment of CAI. It explores the impact of various exercise therapy modalities on this condition and compares their effectiveness to determine which method yields superior results. The control group was rigorously managed, with all included studies maintaining a non-intervention protocol for the control group to eliminate the influence of extraneous factors.

## Methods

### Registration

Meta-analysis establishing the topic was registered (Identifier: CRD42024593686) in the International Prospective Register of Systematic Reviews (PROSPERO).

## Search strategy

Original research articles published from database inception to September 13, 2024, were identified using keywords such as “Exercise Therapy”, “Rehabilitation Exercise”, “Musculoskeletal Manipulations” and “chronic ankle instability” from the following English language literature: PubMed (MEDICINE), Embase, Cochrane library and Web of Science. The inclusion criteria of this meta-analysis included: (a) participants were specifically diagnosed with CAI in patients, (b) intervention group was exercise therapy, (c) comparison group received no intervention, (d) outcomes include quantitative data related to ankle joint function (FAAM and SEBT), (e) the types of studies were RCTs. The exclusion criteria of this meta-analysis included: (a) Patients with non-chronic ankle instability, (b) abstracts, letters editorials, expert opinions, reviews, case reports and laboratory studies, (c) articles written in language other than English editorials.

## Study selection process

Two reviewers independently screened all study titles and abstracts. The full text of the studies that potentially met the inclusion criteria was obtained, and all potentially relevant references were retrieved according to the predefined inclusion criteria. Disagreements were resolved through discussion and, if necessary, consultation with a third investigator to reach a consensus.

## Data extraction

Two reviewers independently extracted data based on the established inclusion and exclusion criteria. RCTs will be divided into two arms according to the interventions: exercise therapy will be considered the exercise group, and control group without any interventions will be designated as the blank group. All outcome parameters included in the meta-analysis are continuous variables. For continuous variables, the mean difference (MD) and its 95% confidence interval (CI) will be used for analysis. If the units of continuous variables differ, the standardized mean difference (SMD) and its 95% CI will be employed instead. If data are missing from the included studies or the results are not reported as means and standard deviation (SD), the authors will be contacted by email for data.

## Assessment of risk of bias and quality

Two reviewers will independently assess the quality of each study using Version 2 of the Cochrane Tool for Assessing Risk of Bias in Randomized Trials (RoB 2)^15^. In cases of disagreement between the two reviewers, a third reviewer will make the final decision.

### Main outcomes

Self-reported ankle instability is assessed using FAAM, which consists of two parts: Foot and Ankle Ability Measure-Activities of Daily Living (FAAM-A) and Foot and Ankle Ability Measure-Sports (FAAM-S). The dynamic stability of the ankle is evaluated using the Star Excursion Balance Test (SEBT). This test measures the ability to maintain balance on one leg while reaching as far as possible with the opposite leg in eight directions: SEBT-anterior (SEBT-A), SEBT-anterolateral (SEBT-AL), SEBT-anteromedial (SEBT-AM), SEBT-lateral (SEBT-L), SEBT-medial (SEBT-M), SEBT-posterior (SEBT-P), SEBT-posteromedial (SEBT-PM), and SEBT-posterolateral (SEBT-PL).

## Study quality

The overall level of evidence was determined using the GRADE Profiler 3.0 software, and the results were categorized into “High,” “Moderate,” “Low,” and “Very Low” for the assessment of main outcomes^16^.

### Statistical analysis

All data analysis will be conducted using the Cochrane Collaboration Review Manager software (RevMan version 5.3.0) and Stata 18.0 software. All outcome parameters included in the meta-analysis are continuous variables. For continuous variables, the MD and its 95%CI will be used for analysis. If the units of continuous variables differ, the SMD and its 95% CI will be employed instead. Heterogeneity will be assessed using the I² statistic. If I² < 50%, a fixed-effects model will be used for analysis. If I² ≥ 50%, a random-effects model will be applied. In multi-arm RCTs, the effect sizes of the exercise therapy group should be combined. Sensitivity analysis was conducted when heterogeneity was high (I² > 50%). To investigate the sources of heterogeneity, a subgroup analysis and meta-regression analysis (with more than 10 studies included) was performed. When the analysis includes more than 10 studies, a funnel plot analysis will be conducted to detect publication bias. Statistical significance will be set at *p =* 0.05.

## Results

### Study selection

**Fig. 1:**
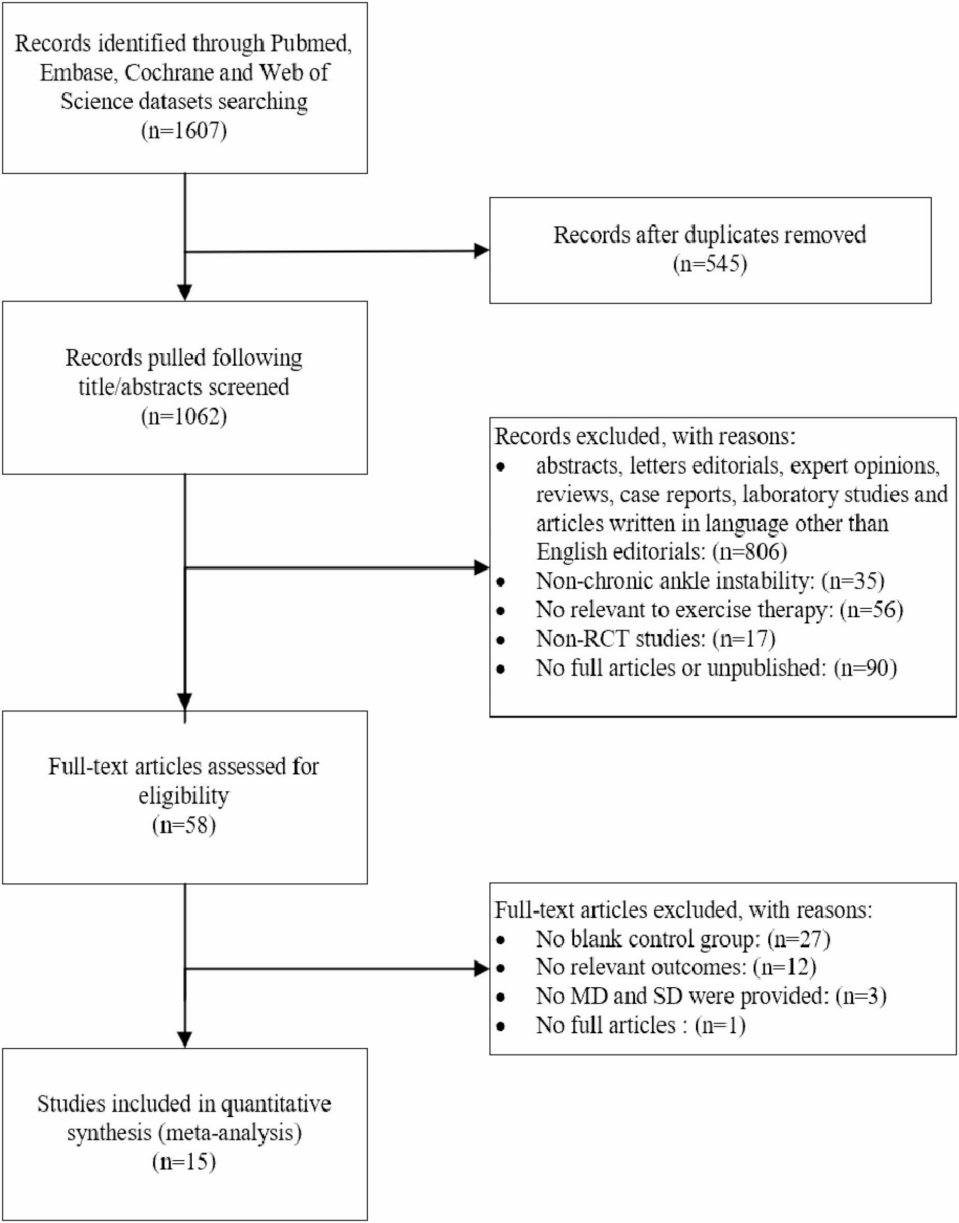
Flowchart of the selection process.

A total of 1,607 articles were retrieved from the database. After removing duplicates, 1,062 articles remained. Title and abstract screening further reduced this number to 58 articles, which underwent full-text screening. Ultimately, 16 articles met the inclusion criteria and were incorporated into our study. The flowchart and reasons for this process are illustrated in Fig. 1.

### Study characteristics

This meta-analysis included a total of 15 studies, comprising patients with CAI. The basic characteristics of the included studies are presented in Table 1.

**Table 1:** Main characteristics of the included studies EG: exercise therapy group, CG: no intervention group.

First author, year	Age (years) Mean ± SD	Sample size	Intervention	Outcome	Treatment duration
Ardakani, 2019	EG: 22.78 ± 3.09 CG: 22.57 ± 2.76	EG: 14 CG:14	EG: Hopping exercises, 3 times per week for 6weeks CG: No intervention	FAAM-A FAAM-S	6 weeks
Beazell, 2014	EG1: 25.2 ± 8.2 EG2: 27.5 ± 8.8 CG: 23.8 ± 5.6	EG1:15 EG2:14 CG: 13	EG1: Proximal Tibiofibular Joint Manipulation, 1–2 min per session, once daily EG2: Distal Tibiofibular Joint Manipulation, 1–2 min per session, once daily CG: No intervention	FAAM-S	3 weeks
Cain, 2017	EG: 16.45 ± 0.93 CG: 16.55 ± 1.29	EG: 11 CG: 11	EG: A progressive Biomechanical Ankle Platform System (BAPS) board rehabilitation program with five difficulty levels, 3 times per week for 4 weeks CG: no intervention	SEBT-AM SEBT-M SEBT-PM	4 weeks
Cain, 2020	EG1: 16.42 ± 1.00 EG2: 16.40 ± 0.97 CG 16.45 ± 1.04	EG1: 12 EG2: 10 CG: 11	EG1: 3 sets of 10 repetitions of ankle plantar flexion, dorsiflexion, inversion, and eversion with a resistance band, 3 times per week for 4 weeks EG2: Biomechanical Ankle Platform System (BAPS) training with five difficulty levels, 3 times per week for 4 weeks CG: No intervention	SEBT-A SEBT-AM SEBT-M SEBT-PM SEBT-PL FAAM-A FAAM-S	4 weeks
Chang, 2021	EG1: 20.31 ± 1.28 EG2: 20.43 ± 1.25 CG: 21.23 ± 1.47	EG1: 21 EG2: 21 CG: 21	EG1: Whole-body vibration (WBV) training, 3 × 30 min/week for 6weeks EG2: Balance training of three exercise movements on the balance ball, 3 × 30 min/week for 6weeks CG: No intervention	SEBT-A SEBT-AL SEBT-AM SEBT-PM SEBT-P SEBT-PL SEBT-M SEBT-L	6 weeks
Cruz-Díaz, 2014	EG: 26.83 ± 4.62 CG: 26.48 ± 4.03	EG: 30 CG: 21	EG: Joint Mobilizations, twice per week for 3 weeks CG: No intervention	SEBT-A SEBT-PM SEBT-PL	3 weeks
Cruz-Díaz, 2020	EG: 35,4 ± 10,46 CG: 36,3 ± 11,98	EG: 25 CG: 24	EG: Tai Chi training, 2 × 60 min/week for 12weeks CG: No intervention	SEBT-A SEBT-PM SEBT-PL	12 weeks
Harkey, 2014	EG: 21.5 ± 3.4 CG: 20.8 ± 2.0	EG: 15 CG: 15	EG: Joint Mobilization, 1 time CG: No intervention	SEBT-A SEBT-PM SEBT-PL	1 time
Lapanantasin, 2022	EG1: 19.5 ± 1.1 EG2: 19.9 ± 0.9 CG: 19.6 ± 1.3	EG1:10 EG2:11 CG:11	EG1: Walking meditation, 3 × 30 min/week for 4weeks EG2: Rubber-band exercise, 3 × 3 × 15 times per week for 4 weeks CG: No intervention	SEBT-A SEBT-AL SEBT-L SEBT-PL SEBT-P SEBT-PM SEBT-M SEBT-AM	4 weeks
Linens, 2016	EG: 22.94 ± 2.77 CG: 23.18 ± 3.64	EG: 17 CG: 17	EG: Wobble board rehabilitation with five difficulty levels, 3 times per week for 4 weeks CG: No intervention	SEBT-AM SEBT-M SEBT-PM	4 weeks
Mckeon, 2008	EG: 22.2 ± 4.5 CG: 19.5 ± 1.2	EG: 16 CG: 15	EG: Progressive balance-training program with seven difficulty levels, 3 × 20 min/week for 4weeks CG: No intervention	SEBT-A SEBT-PM SEBT-PL	4 weeks
Mckeon, 2016	EG1: 23.6 ± 6.7 EG2: 22.3 ± 2.7 EG3: 22.0 ± 2.8 CG: 22.9 ± 4.5	EG1: 20 EG2: 20 EG3: 20 CG: 20	EG1: 2 sets of two-minutes of Grade III anterior-to-posterior talocrural joint mobilizations, with a one-minute rest between sets, once a day for two weeks EG2: Two-minute plantar massage sets with a one-minute rest between sets, once a day for two weeks EG3: Two sets of triceps surae stretching, 3 × 30s/times, once a day for two weeks CG: No intervention	FAAM-A FAAM-S	2 weeks
Reyes, 2024	EG: 22.7 ± 3.3 CG: 22.3 ± 3.1	EG:15 CG:15	EG: Unilateral Balance training, 3 × 20 min per week for 8 weeks CG: No intervention	SEBT-A SEBT-PM SEBT-PL	8 weeks
Smith, 2018	EG: 20.1 ± 1.69 CG: 20.9 ± 1.26	EG: 13 CG: 13	EG: 5-minute general body warm-up and 3 sets of 20 repetitions of progressive resistance exercises, 3 times a week for 4 weeks CG: No intervention	SEBT-A SEBT-PM SEBT-PL FAAM-A FAAM-S	4 weeks
Thanasootr, 2022	EG: 22.89 (5.60) CG: 23.89 (6.58)	EG: 9 CG:9	EG: Nine-square exercise, 30 min of nine-square exercise and 30 min of warm-up and cool-down, 18 times over the 6 weeks CG: No intervention	FAAM-A FAAM-S	6 weeks

### Risk of bias assessment

**Fig. 2:**
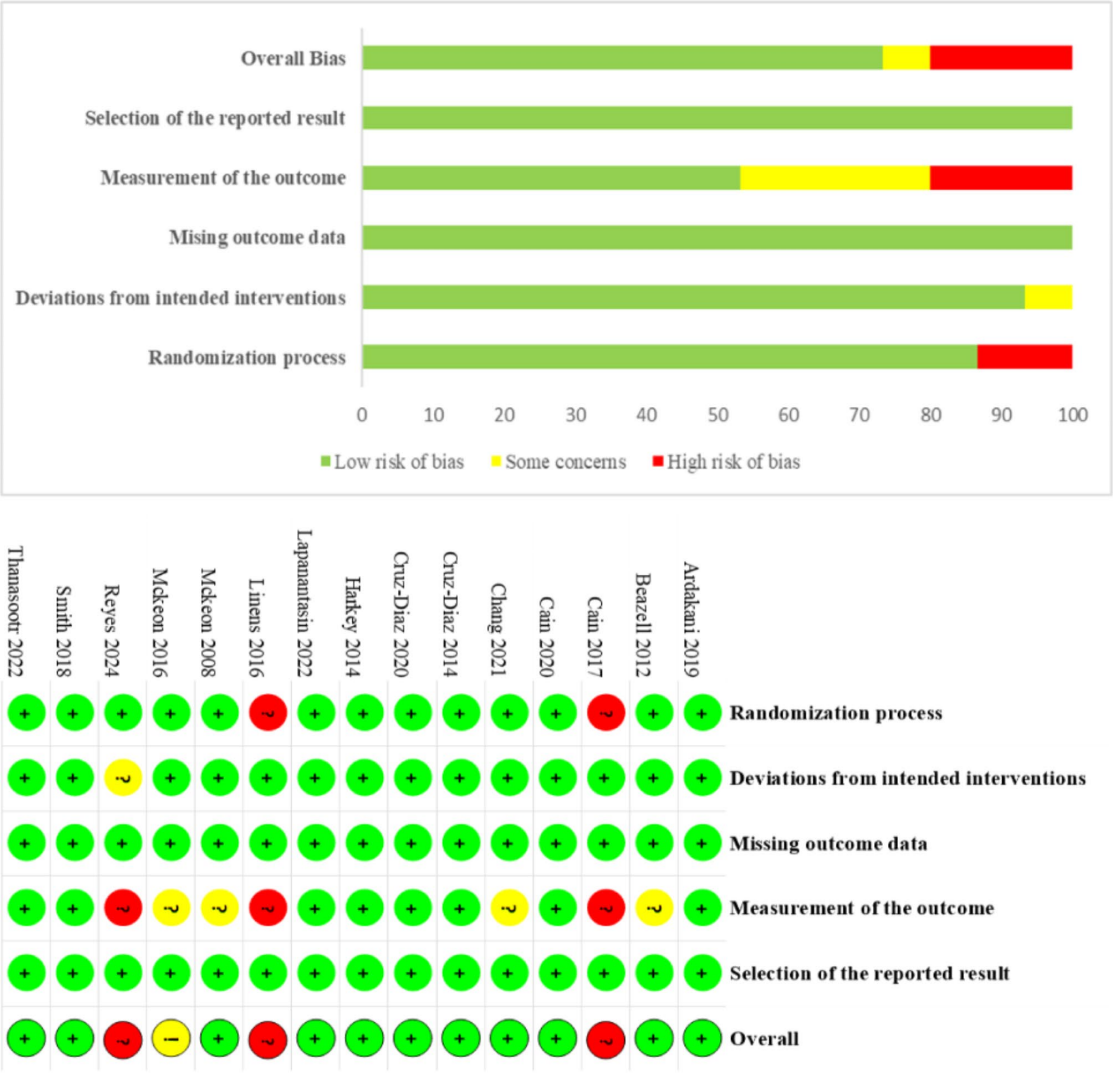
Risk of bias summary and graph.

Using RoB 2 to assess the methodological quality of all included studies. In Domain 1: Randomization Process, 13 articles provided a detailed description of the randomization methods and were rated as having a low risk of bias^17–29^. In contrast, two articles did not specify the randomization process or provide a registration number for the randomized controlled clinical trial, resulting in a high risk of bias^30,31^. In Domain 2: Deviations from Intended Interventions, 14 studies were assessed as having a low risk of bias[^17–26,28–31^]. However, one study raised some concerns due to the inclusion of patients with bilateral CAI, which may impact the results^27^. In Domain 3: Missing Outcome Data, all articles provided specific data and were assessed as having a low risk of bias. In Domain 4: Measurement of the Outcome, eight studies explicitly stated that outcome assessors were blinded, and these were rated as having a low risk of bias^17,19,21–24,28,29^. Three studies were rated as raising some concerns^18,20,25^. Three studies were assessed as having a high risk of bias due to the lack of blinding for outcome assessors^27,30,31^. One study, although lacking blinding of outcome assessors, utilized multiple assessment methods and was rated as raising some concerns^26^. In Domain 5: Selection of the Reported Result, all articles were assessed as having a low risk of bias. Overall, 11 studies were rated as low risk^17–25,28,29^, one study raised some concerns^26^, and three studies were assessed as having a high risk of bias^27,30,31^. These results are summarized in Fig. 2.

### Meta‑analysis results

#### Meta‑analysis results of exercise therapy on self‑functional scores of patients with CAI

A total of six studies were included that examined the impact of exercise therapy on self-reported functional assessment in patients with CAI, utilizing FAAM-A and FAAM-S as assessment tools.

**Fig. 3:**
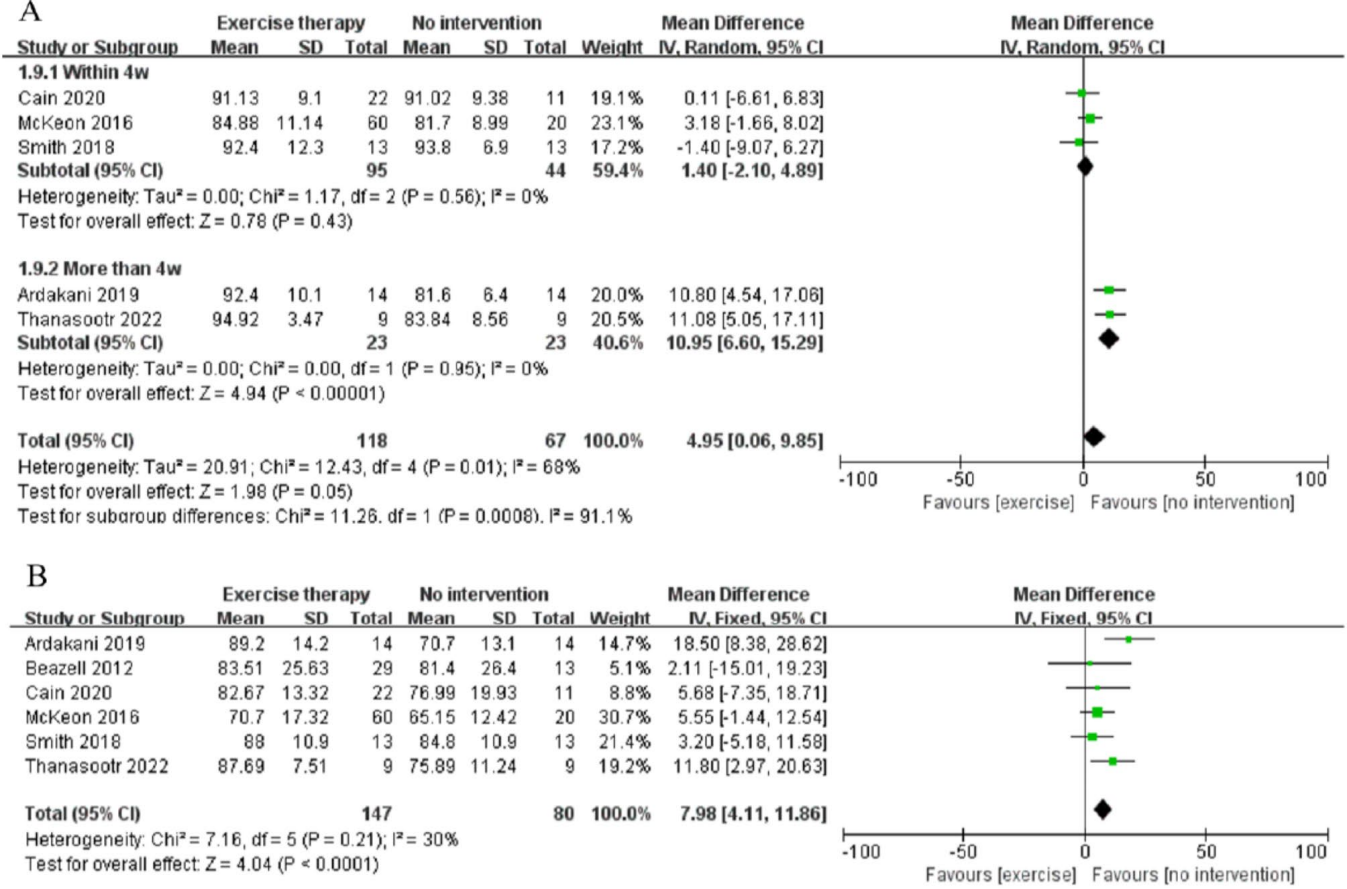
Meta‑analysis results of exercise therapy on self-functional scores of patients with CAI, A: FAAM-A, B: FAAM-S.

Five studies analyzed FAAM-A, involving 185 patients with CAI (exercise therapy group: 118; control group: 67)^17,19,26,28,29^. The pooled results indicated a significant effect of exercise therapy on FAAM-A (MD = 4.95, CI: 0.06 to 9.85, *p* = 0.05, I² = 68%) (Fig. 3A). Due to high heterogeneity (I² = 68%), a sensitivity analysis was conducted. However, regardless of which study was excluded, high heterogeneity persisted (I² > 50%). The GRADE of evidence was categorized as low quality because of high heterogeneity (I² > 50%) among studies and insufficient sample sizes (*n* < 200).

To investigate the sources of heterogeneity, a subgroup analysis was performed based on the intervention period, categorizing it into ≤ 4 weeks and > 4 weeks. After subgrouping by intervention duration, no significant heterogeneity was observed in either the subgroup of less than 4 weeks (I² = 0) or the subgroup of more than 4 weeks (I² = 0), indicating that the intervention duration was a source of heterogeneity. The subgroup analysis indicated that interventions lasting more than 4 weeks yielded better outcomes (MD = 10.95, CI: 6.60 to 15.29, *p* < 0.00001, I² = 0%) (Fig. 3A), whereas interventions within 4 weeks showed no significant difference compared to the control group (MD = 1.4, CI: -2.10 to 4.89, *p* = 0.43, I² = 0%).

Six studies analyzed FAAM-S, including 227 patients with CAI (exercise therapy group: 147; control group: 80)^17–19,26,28,29^. The pooled results demonstrated a significant improvement in FAAM-S scores due to exercise therapy (MD = 7.98, CI: 4.11 to 11.86, *p* < 0.0001, I² = 30%) (Fig. 3B).

### Meta‑analysis results of exercise therapy on dynamic balance ability of patients with CAI

SEBT consists of assessments in eight directions. A total of 11 studies were included in the analysis of the SEBT, which comprised the following: SEBT-A (9 studies)^19–25,27,28^, SEBT-AL (2 studies)^20,24^, SEBT-AM (4 studies)^19,20,24,31^, SEBT-L (2 studies)^20,24^, SEBT-M (4 studies)^19,20,24,31^, SEBT-P (2 studies)^20,24^, SEBT-PM (11 studies)^19–25,27,28,30,31^, and SEBT-PL (9 studies)^19–25,27,28^.

**Fig. 4:**
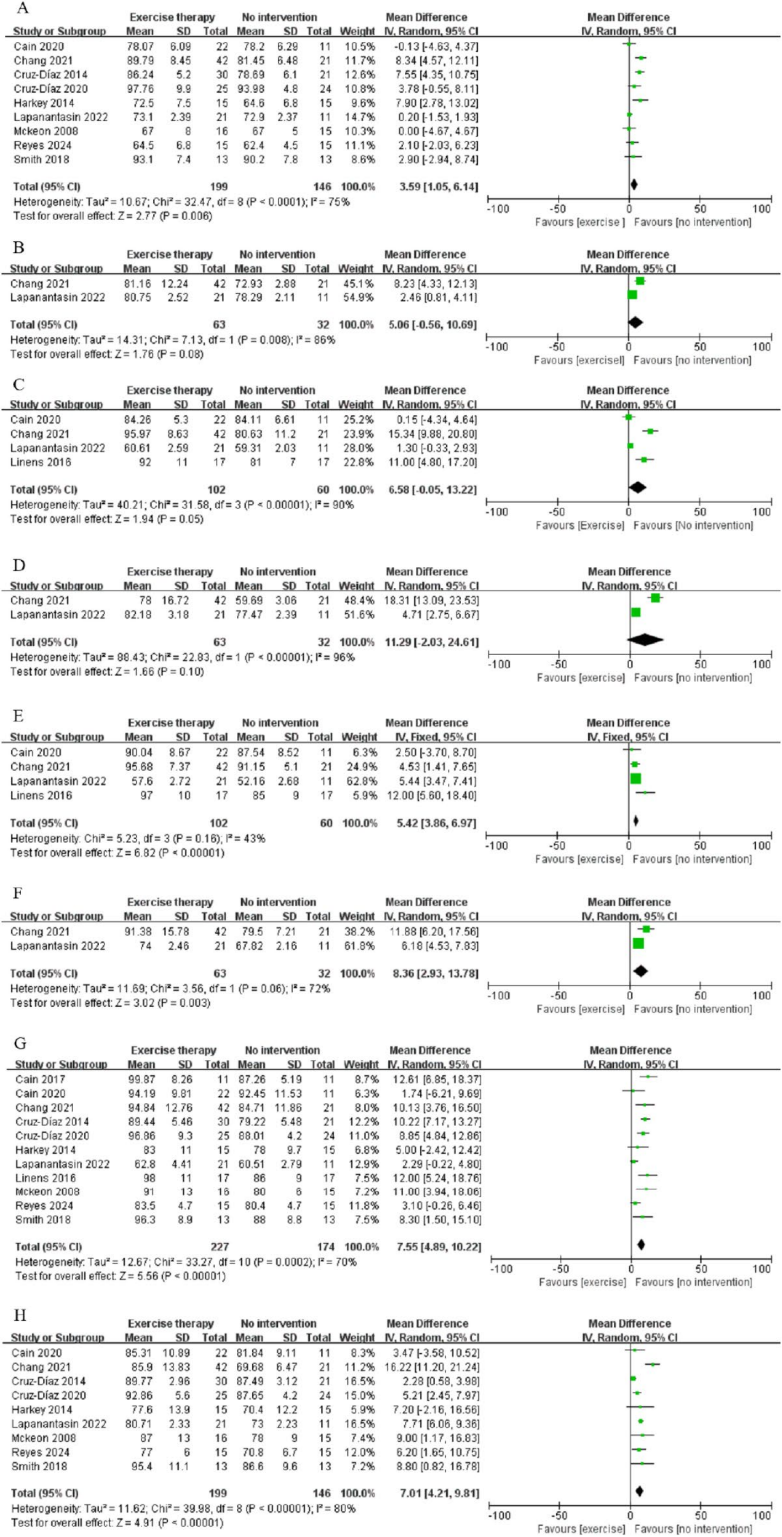
Meta‑analysis results of exercise therapy on dynamic balance ability of patients with CAI, A: SEBT-A, B: SEBT-AL, C: SEBT-AM, D: SEBT-L, E: SEBT-M, F: SEBT-P, G: SEBT-PM, H: SEBT-PL.

The results were summarized in Fig. 4. The findings indicated that exercise therapy significantly improved in SEBT-A (MD = 3.59, CI: 1.05 to 6.13, *p* = 0.006, I² = 75% ) (Fig. 4A), SEBT-AM (MD = 6.58, CI: -0.05 to 13.22, *p* = 0.05, I² = 90%) (Fig. 4C), SEBT-M (MD = 5.42, CI: 3.86 to 6.97, *p* < 0.00001, I² = 43%) (Fig. 4E), SEBT-P (MD = 8.36, CI: 2.93 to 13.78, *p* = 0.003, I² = 72%) (Fig. 4F), SEBT-PM (MD = 7.55, CI: 4.89 to 10.22, *p* < 0.00001, I² = 70%) (Fig. 4G), and SEBT-PL (MD = 7.01, CI: 4.22 to 9.81, *p* < 0.0001, I² = 80%) (Fig. 4H). There were no significant effects on SEBT-AL (MD = 5.06, CI: -5.06 to 10.69, *p* = 0.08, I² = 86%) (Fig. 4B) and SEBT-L (MD = 11.29, CI: -2.03 to 24.61, *p* = 0.10, I² = 96%) (Fig. 4D).

Due to the high heterogeneity observed in the heterogeneity tests (I² > 50%), a sensitivity analysis was conducted by sequentially excluding the included studies. For SEBT-AL, SEBT-L, and SEBT-P, only two studies were included, rendering the sensitivity analysis meaningless. For SEBT-AM, the results became non-significant after the exclusion of two studies, indicating that the included studies had an unstable impact on the results for SEBT-AM. For SEBT-M, the removal of Linens et al. study resulted in the disappearance of heterogeneity (I² = 0%). For SEBT-A, SEBT-PM, and SEBT-PL, high heterogeneity persisted regardless of which study was excluded, with the exercise therapy group consistently outperforming the control group (*p* < 0.05).

To investigate the sources of heterogeneity, Meta-regression and subgroup analysis were conducted. Three subgroups were defined based on the included studies: countries, intervention period and type of exercise therapy. Countries were categorized into the United States, China, Germany and Thailand. The intervention period was categorized into ≤ 4 weeks and > 4 weeks, while the type of exercise therapy included strength training, neuromuscular activation, proprioceptive training and joint mobilization. The classification of exercise modalities was established based on the descriptions of therapeutic interventions provided in included studies. For instance, Cain et al.^30^ emphasized the importance of proprioceptive training utilizing the Biomechanical Ankle Platform System (BAPS), while Chang et al.^20^ proposed that Whole-body vibration (WBV) training enhances α motor neuron excitability and improves the synchronization of motor units.

### Results of the meta-regression analysis

Meta-regression analysis was performed to evaluate the sources of heterogeneity. The results showed that the between-subgroup differences were not significant in any of the subgroups (*p* > 0.05), as presented in Table 2. In the country subgroup, there was only one study each from China, Germany, and Thailand, while most studies were from the United States, making comparisons less meaningful. Therefore, we selected intervention duration and exercise type as subgroups for further subgroup analysis.

**Table 2:** Result of Meta-regression analysis.

subgroup		Coefficient	Standard Error.	t	*P* > | t |	95% CI
countries	SEBT-A	-1.479	1.066	-1.39	0.208	-4.000, 1.042
	SEBT-PM	-2.567	1.178	-2.18	0.057	-5.232, 0.0987
	SEBT-PL	0.287	1.511	0.19	0.855	-3.285, 3.859
intervention period	SEBT-A	1.846	2.642	0.7	0.507	-4.401, 8.093
	SEBT-PM	-0.852	2.895	-0.29	0.775	-7.4, 5.697
	SEBT-PL	2.833	3.06	0.93	0.385	-4.401,10.068
type of exercise therapy	SEBT-A	0.605	1.097	0.55	0.593	-1.84, 3.05
	SEBT-PM	0.929	1.268	0.73	0.478	-1.835, 3.693
	SEBT-PL	-2.461	1.19	-2.07	0.066	-5.113, 0.191

### Results of the subgroup analysis

The results of the subgroup analysis are shown in Table 3.

**Table 3:** Result of subgroup analysis on SEBT of patients with CAI.

Regulatory variables	Subgroup	Number of effectors	SEBT-A MD (95%CI)	SEBT-PM MD (95%CI)	SEBT-PL MD (95%CI)
Intervention period	≤ 4 weeks	20	2.98 (-0.27, 6.23)	7.86 (4.29, 11.42)	5.89 (2.51, 9.27)
	> 4 weeks	9	4.83 (1.04, 8.63)	6.93 (2.37, 11.48)	8.98 (2.66, 15.29)
Types of Exercise Therapy	Strength Training	12	3.69 (-1.39, 8.77)	7.63 (3.37, 11.88)	9.52 (7.56, 11.47)
	Neuromuscular activation	12	2.62 (-0.01, 5.25)	4.00 (-0.64, 8.64)	6.56 (4.91, 8.21)
	Proprioceptive Training	7	-0.94 (-4.66, 2.79)	10.46 (5.27, 15.65)	6.40 (0.41, 12.38)
	Joint Mobilization	6	7.65 (4.93, 10.37)	8.75 (4.15, 13.35)	2.44 (0.76, 4.11)

### Intervention period subgroup

For the intervention period of ≤ 4 weeks, significant improvements were observed in SEBT-PM (MD = 7.85, CI: 4.29 to 11.42, *p* < 0.0001, I² = 74%)和SEBT-PL (MD = 5.89, CI: 2.50 to 9.27, *p* = 0.0007, I² = 78%); however, no improvement was noted in SEBT-A (MD = 2.99, CI: -0.27 to 6.24, *p* = 0.07, I² = 78%). For the intervention period of > 4 weeks, improvements were seen across all three directions of SEBT: SEBT-A (MD = 4.83, CI: 1.04 to 8.63, *p* = 0.01, I² = 62%), SEBT-PM (MD = 6.93, CI: 2.37 to 11.48, *p* = 0.003, I² = 69%) and SEBT-PL (MD = 8.98, CI: 2.66 to 15.29, *p* = 0.005, I² = 86%). These results suggest that interventions lasting more than 4 weeks may lead to better improvements in SEBT outcomes for patients with CAI. Due to the high heterogeneity among subgroups, the conclusions should be interpreted with caution.

### Sensitivity analysis of intervention period subgroup

Due to the high heterogeneity within subgroups, sensitivity analysis was further conducted. The results demonstrated that: after excluding the study by Cruz-Diaz et al. (2014), the heterogeneity of SEBT-PL (≤ 4 weeks) disappeared (I² = 0, *p* < 0.00001); after excluding the study by Chang et al. (2021), the heterogeneity of both SEBT-A (> 4 weeks) and SEBT-PL (> 4 weeks) disappeared (I² = 0, *p* = 0.06; I² = 0, *p* < 0.00001); after excluding the study by Lapanantasin et al. (2022), the heterogeneity of SEBT-PM (≤ 4 weeks) disappeared (I² = 17%, *p* < 0.00001), and the heterogeneity of SEBT-PL (≤ 4 weeks) significantly decreased (I² = 31%, *p* = 0.004); after excluding the study by Reyes et al., the heterogeneity of SEBT-PM (> 4 weeks) disappeared (I² = 0, *p* < 0.00001).

### Exercise type subgroup

The subgroup analysis of exercise therapy classifications indicated that strength training resulted in significant improvements in SEBT-PM (MD = 7.63, CI: 3.37 to 11.88, *p* = 0.0004, I² = 47%) and SEBT-PL (MD = 8.15, CI: 6.09 to 10.21, *p* < 0.00001, I² = 0%), but showed no notable difference in SEBT-A (MD = 3.69, CI: -1.39 to 8.77, *p* = 0.15, I² = 82%). Neuromuscular activation resulted in significant improvements in SEBT-PL (MD = 6.56, CI: 4.91 to 8.21, *p* < 0.00001, I² = 0%), but showed no notable difference in SEBT-A (MD = 2.62, CI: -0.01 to 5.25, *p* = 0.05, I² = 43%) and SEBT-PM (MD = 4.00, CI: -0.64 to 8.64, *p* = 0.09, I² = 80%). Proprioceptive training resulted in significant improvements in SEBT-PM (MD = 10.46, CI: 5.27 to 15.65, *p* < 0.0001, I² = 33%) and SEBT-PL (MD = 6.40, CI: 0.41 to 12.38, *p* = 0.04, I² = 2%), but showed no notable difference in SEBT-A (MD = -0.94, CI: -4.66 to 2.79, *p* = 0.15, I² = 82%). Joint mobilization resulted in significant improvements in SEBT-A (MD = 7.65, CI: 4.93 to 10.37, *p* < 0.00001, I² = 0%), SEBT-PM (MD = 8.75, CI: 4.15 to 13.35, *p* = 0.0002, I² = 38%) and SEBT-PL (MD = 2.44, CI: 0.76 to 4.11, *p* = 0.004, I² = 3%).

#### Sensitivity analysis of exercise type subgroup

After subgroup analysis, heterogeneity was eliminated in most subgroups while significant heterogeneity remained in certain subgroups. Sensitivity analysis was conducted to further explore the sources of heterogeneity. For the strength training of SEBT-A and SEBT-PL, the removal of Chang et al. study eliminated within-group heterogeneity (I² = 0), and did not affect the overall results (SBET-A: *p* = 0.54, SEBT-PL: *p* < 0.00001). For the neuromuscular activation of SEBT-PM, the removal of Lapanantasin et al. study significantly reduced within-group heterogeneity (I² = 57%), but the overall results were reversed (*p* = 0.005).

These results indicate that the type of exercise is a source of heterogeneity, and studies by Chang and Lapanantasin et al. also contribute to heterogeneity. However, these sources of heterogeneity have a minimal impact on the overall results. This demonstrates that the findings of our meta-analysis are generally reliable. Nevertheless, caution should be exercised when interpreting the effects of neuromuscular activation on SEBT-PM.

### Efficacy validation of exercise therapy

**Fig. 5:**
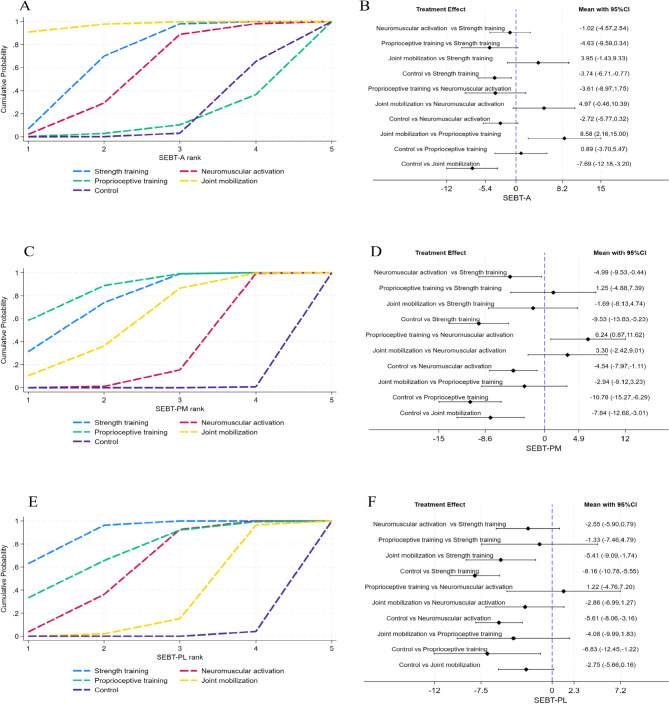
SUCRA plot and pairwise comparison forest plot for the classification of exercise therapy, SUCER plot: A (SEBT-A), C (SEBT-PM), E (SEBT-PL); pairwise comparison forest plot: B (SEBT-A), D (SEBT-PM), F (SEBT-PL).

To further assess which form of exercise therapy has a better efficacy rate, the control group was used as a common reference. Indirect comparisons were conducted for the studies included SEBT-A, SEBT-PM, and SEBT-PL. The Surface Under the Cumulative Ranking curve (SUCRA) plot and pairwise comparison forest plot are displayed in Fig. 5. The area under the SUCRA curve reflects the efficacy of each therapy, with a larger area indicating greater effectiveness. For SEBT-A, joint mobilization demonstrated the most effective results, and strength training also showed favorable therapeutic effects, with both interventions exhibiting significant differences compared to the control group; the other two treatment methods outperformed the control group, but the differences were not statistically significant (Fig. 5A-B). For SEBT-PM, all treatment methods exhibited good efficacy, with significant differences observed compared to the control group; strength training and proprioceptive training showed superior effects, significantly outperforming the other two groups (Fig. 5C-D). For SEBT-PL, strength training demonstrated the most effective results, followed by proprioceptive training and neuromuscular activation, with these groups showing significant differences compared to the control group; joint mobilization did not demonstrate a significant difference (Fig. 5E-F). Overall, these findings indicate that joint mobilization is the most effective intervention for improving SEBT-A, while strength training and proprioceptive training are the most effective for enhancing SEBT-PL.

#### Evaluation of publication bias in literature

**Fig. 6:**
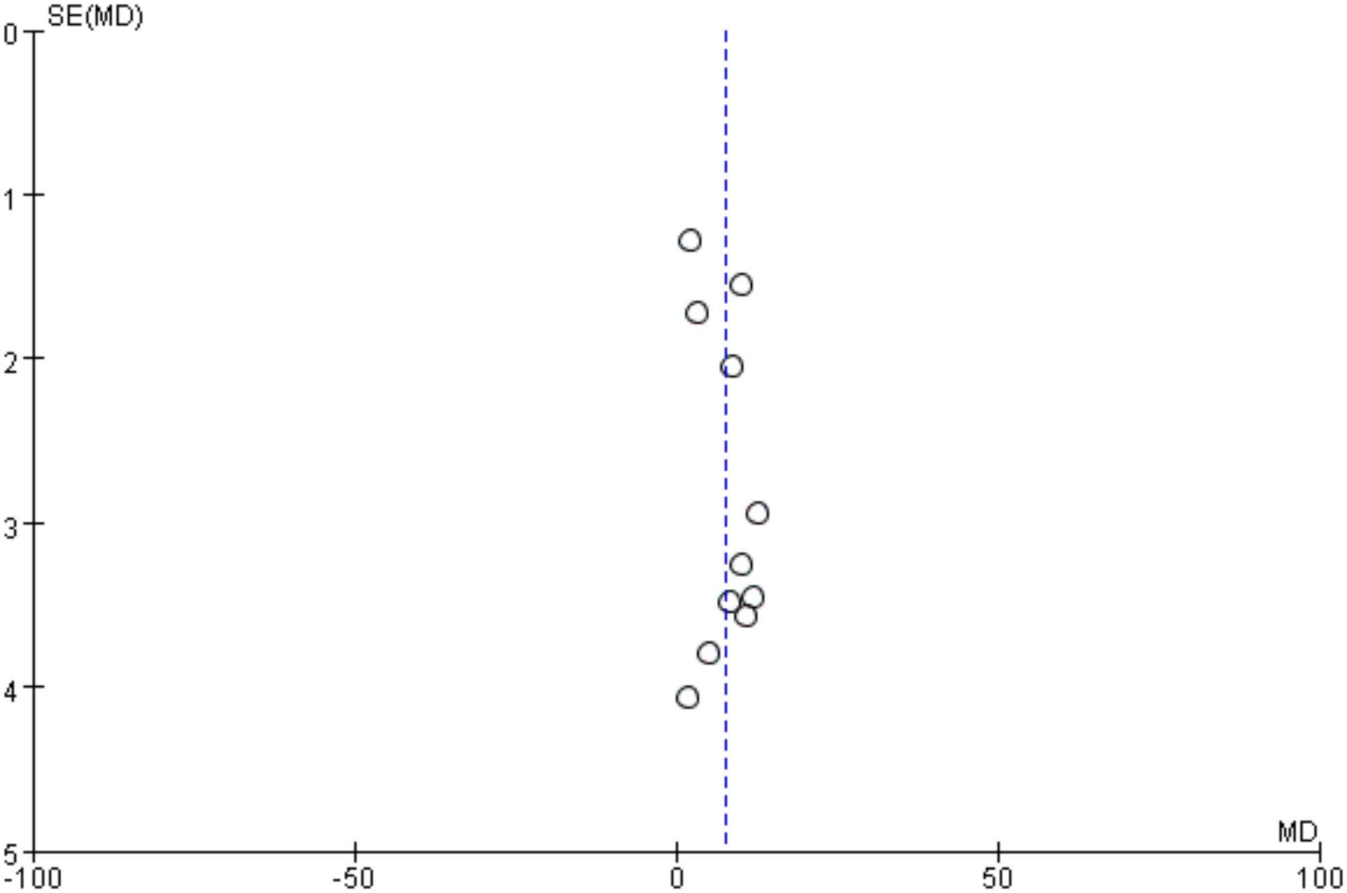
Funnel plot about meta-analysis of SEBT.

Since more than 10 studies were included for SEBT, a funnel plot analysis was conducted to assess publication bias. The funnel plot revealed a generally symmetrical distribution on both sides, indicating that publication bias is unlikely to have a significant impact (Fig. 6).

#### Quality of evidence (GRADE)

The quality levels of the results analyzed using the GRADE approach are illustrated in Table 4. Following the GRADE scoring principles, the overall quality of evidence for the six efficacy outcomes was rated as low or very low, primarily due to high heterogeneity among studies and insufficient sample sizes.

**Table 4:** GRADE evidence profile table for CAI.

No of studies	Risk of bias	Inconsistency	Indirectness	Imprecision	Publication bias	Relative (95% CI)	Quality	Importance	
FAAM-A (5)	No	Serious^1^	No	No	No	4.95 (0.06 to 9.85)	Moderate	Critical	
FAAM-S (6)	No	no	No	No	No	7.98 (4.11 to 11.86)	High	Critical	
SEBT-A (9)	No	Very serious^2^	No	No	No	3.59 (1.05 to 6.14)	Low	Critical	
SEBT-AL (2)	No	Very serious^2^	No	Very serious^3,4^	No	5.06 (-0.56 to 10.69)	Very Low	Important	
SEBT-AM (6)	No	Very serious^2^	No	Serious^3^	No	4.58 (0.34 to 8.81)	Very Low	Important	
SEBT-M (6)	No	No	No	Serious^3^	No	5.42 (3.87 to 6.97)	Moderate	Important	
SEBT-PM (11)	No	Serious^1^	No	No	No	7.55 (4.89 to 10.22)	Moderate	Critical	
SEBT-PL (9)	No	Very serious^2^	No	No	No	7.01 (4.21 to 9.81)	LOW	Critical	
SEVT-L (2)	No	Very serious^2^	No	Serious^3^	No	11.29 (-2.03 to 24.61)	VERY LOW	Important	
SEBT-P (2)	No	Serious^1^	No	Serious^3^	No	8.36 (2.93 to 13.78)	LOW	Important	

^1^ Heterogeneity greater than 50%.

^2^ Heterogeneity greater than 75%.

^3^ The sample size included in the study was small (*n* < 180).

^4^ The confidence interval is relatively wide.

## Discussion

CAI is often caused by injuries from physical activities, but the exact mechanisms are not well understood. Research indicates that changes in frontal plane movement and decreased peroneal activation after ankle sprains may contribute to this condition^32^. Exercise therapy serves as the primary treatment modality, enhancing ankle proprioception and muscle strength, thereby helping to reduce the risk of re-injury^33^. The aim of this meta-analysis is to evaluate the efficacy of exercise therapy in the treatment of CAI, explore the optimal treatment approaches, and provide clinical evidence for the development of CAI rehabilitation strategies.

FAAM is a reliable self-assessment tool with two components: FAAM-A, which evaluates daily activity performance, and FAAM-S, which assesses sports performance^34^. Our findings indicate that exercise therapy has a significant improvement in FAAM scores, with a notably greater improvement observed in FAAM-S, consistent with the findings reported by Guo et al.^35^. The limited emphasis on daily living activities in the treatment protocols of the included studies may explain this observation. However, the results for FAAM-A show significant heterogeneity, indicating differences among the studies. Subgroup analysis based on intervention duration revealed that longer interventions (exceeding 4 weeks) yielded better outcomes, likely due to the increased stability of treatment effects over extended training periods. Following subgroup analysis, heterogeneity within each subgroup was eliminated, indicating that intervention duration is a key factor contributing to between-study heterogeneity and supporting the reliability of our results. These findings suggest that long-term exercise therapy is effective in improving daily function and performance in patients with CAI. The FAAM outcomes demonstrated moderate to high GRADE evidence levels, reflecting strong reliability of the findings. Future research should focus on intervention duration to optimize treatment protocols and enhance recovery.

SEBT is a dynamic balance assessment method that effectively evaluates balance and coordination, making it suitable for sports rehabilitation and functional assessment^36^. Y-Balance Test is a modified version of the SEBT, focusing on forward, posterolateral, and posteromedial extensions, which simplifies the testing process^37^. In the evaluation of the SEBT, most studies that rely on Y-Balance Test have selected SEBT-A, SEBT-PL, and SEBT-PM as the primary outcome measures. SEBT-AM, SEBT-M, and SEBT-PM are considered the best for distinguishing healthy individuals from those with ankle instability^38^. While studies using SEBT-AM and SEBT-M have shown good results, only four exist, making it difficult to confirm efficacy. Research on SEBT-P, SEBT-AL, and SEBT-L is even more limited, with just two studies. Different outcome measures may affect treatment results and introduce selection bias.

The sensitivity analysis showed high heterogeneity in SEBT-A, SEBT-PL, and SEBT-PM, but excluding any single study did not significantly alter the overall results. Subgroup analysis revealed that treatments lasting over four weeks yielded better outcomes, consistent with FAAM results. However, due to unexplained high heterogeneity of uncertain causes, we need to be cautious of this result.

The results of meta-regression and sensitivity analyses indicate that exercise type and two specific studies are the primary sources of heterogeneity. Excluding these studies significantly reduced or eliminated heterogeneity in some subgroups. These findings highlight the critical role of exercise type in CAI rehabilitation and provide valuable insights for clinical practice and experimental design. Additionally, the geographical distribution of participants—with two studies conducted in China^20^ and Thailand^24^, and the majority of others in the United States (except one in Iran)—may reflect differences in participant characteristics, further explaining the observed heterogeneity.

In the subgroup analysis of exercise therapy types, the effect sizes of the two groups cannot be directly combined due to differing interventions in the two multi-arm RCTs. Instead, we will categorize samples by exercise type and evenly allocate the control group samples based on the number of intervention groups. This approach is intended to prevent the repeated inclusion of the control group from affecting the analysis results. After classification, the within-group heterogeneity significantly decreased, indicating that the type of exercise may be a source of heterogeneity. The GRADE evidence levels for these outcomes were moderate to low, mainly due to high heterogeneity, potentially affecting reliability. Meta-regression analysis and heterogeneity analysis showed high heterogeneity persisted in the intervention duration subgroup but resolved in the exercise type subgroup. Thus, the evidence primarily impacts the intervention duration subgroup, while the exercise type subgroup results remain highly reliable. However, these results are not enough to determine the optimal efficacy of different exercise types.

To identify the most effective treatment method and guide exercise prescriptions, we conducted indirect comparisons of the four exercise types, using the control group as a common reference. Different exercise therapies exhibit distinct advantages in improving balance capacity across various directions: strength training demonstrates the most effective overall results for SEBT, particularly excelling in enhancing SEBT-PL; joint mobilization shows more pronounced improvements in SEBT-A, likely due to the anterior-to-posterior talar mobilizations employed in the studies; and proprioceptive training is more effective in improving SEBT-PM. Recent evidence suggests that a multifaceted rehabilitation approach is the most effective strategy for CAI management, which aligns with the findings of our study^39^.

This meta-analysis has several limitations: First, due to the nature of the interventions, patients inevitably know which treatment they are receiving, which may influence their self-reported functional scores and introduce bias. Second, most of the studies did not provide follow-up data on participants, leaving us unclear about the recurrence of CAI. The lack of subsequent data hinders our ability to comprehensively assess the durability and stability of treatment outcomes. Third, this analysis only included RCTs with a control group that received no intervention, excluding other relevant studies. This may limit our conclusions about the best treatment methods. To strengthen the evidence, future more large-scale RCTs incorporating active control groups (such as conventional physiotherapy, orthotic interventions, or multimodal approaches) are needed to enable comprehensive comparisons and enhance the clinical applicability of the finding.

## Conclusion

For patients with CAI, Long-term and multifaceted exercise therapy yields superior rehabilitation outcomes, providing critical clinical evidence for optimizing personalized rehabilitation programs. Clinically, the most suitable exercise therapy can be selected based on SEBT assessment results to develop targeted treatment plans. Specifically, if the SEBT assessment identifies an anterior balance deficit (SEBT-A), joint mobilization is prioritized as the primary intervention. Conversely, in cases of impaired posterolateral stability (SEBT-PL) and diminished posteromedial reach (SEBT-PM), a combined therapeutic approach incorporating strength training and proprioceptive training is recommended. However, given the high heterogeneity observed in the evidence for SEBT indicators (SEBT-A, SEBT-PL, and SEBT-PM), definitive conclusions cannot be drawn at this time, and the findings should be interpreted with caution. Current research still lacks long-term follow-up data and direct comparisons of different exercise therapies for CAI. Future studies should focus on evaluating the durability of exercise therapy and further explore the specific effects of different exercise interventions to optimize rehabilitation protocols.

## Electronic supplementary material

Below is the link to the electronic supplementary material.

Supplementary Material 1

## Data Availability

The datasets generated or analyzed during this study are available from the corresponding author on reasonable request.
