# Supplementary material for: Effectiveness of exercise therapy on chronic ankle instability: a meta-analysis
Source: Sci Rep. 2025 Apr 5;15:11709. doi: 10.1038/s41598-025-95896-w (PMC11972327; doi:10.1038/s41598-025-95896-w)
Supplement: Supplementary file 1 — Supplementary Material 1 [file 41598_2025_95896_MOESM1_ESM.docx]

Pubmed, Cochrance and Embase : (((((((((((((Craniosacral Massage) OR (Reflexology)) OR (Manipulation Therapy)) OR (Manipulation Therapies)) OR (Manipulative Therapies)) OR (Manipulative Therapy)) OR (Manual Therapies)) OR (Manual Therapy)) OR (Bodywork)) OR (Bodyworks)) OR (Rolfing)) OR ("Musculoskeletal Manipulations"[Mesh])) OR ((((((Rehabilitation Exercise) OR (Rehabilitation Exercises)) OR (Exercise Therapies)) OR (Remedial Exercise)) OR (Remedial Exercises)) OR ("Exercise Therapy"[Mesh]))) AND (chronic ankle instability)

WOS:TS=(Craniosacral Massage OR Reflexology OR Manipulation Therapy OR Manipulation Therapies OR Manipulative Therapies OR Manipulative Therapy OR Manual Therapies OR Manual Therapy OR Bodywork OR Bodyworks OR Rolfing OR Musculoskeletal Manipulations) OR TS=(Rehabilitation Exercise OR Rehabilitation Exercises OR Exercise Therapies OR Remedial Exercise OR Remedial Exercises OR Exercise Therapy） and TS=chronic ankle instability
